# Supplementary material for: OpenMM 7: Rapid development of high performance algorithms for molecular dynamics
Source: PLoS Comput Biol. 2017 Jul 26;13(7):e1005659. doi: 10.1371/journal.pcbi.1005659 (PMC5549999; doi:10.1371/journal.pcbi.1005659)
Supplement: S1 Source Code — (ZIP) [file pcbi.1005659.s002.zip › OpenMM-7.0.1-Source/docs/api-python/app.html]

Application Layer — OpenMM 7.0.1.dev-5e86c4f documentation


# Application Layer¶

## Loaders and Setup¶

|  |  |
| --- | --- |
| `AmberInpcrdFile`(file[, loadVelocities, ...]) | AmberInpcrdFile parses an AMBER inpcrd file and loads the data stored in it. |
| `AmberPrmtopFile`(file) | AmberPrmtopFile parses an AMBER prmtop file and constructs a Topology and (optionally) an OpenMM System from it. |
| `CharmmCrdFile`(fname) | Reads and parses a CHARMM coordinate file (.crd) into its components, namely the coordinates, CHARMM atom types, resid, resname, etc. |
| `CharmmPsfFile`(\*args, \*\*kwargs) | A chemical structure instantiated from CHARMM files. |
| `CharmmRstFile`(fname) | Reads and parses data, velocities and coordinates from a CHARMM restart |
| `DCDFile`(file, topology, dt[, firstStep, ...]) | DCDFile provides methods for creating DCD files. |
| `DesmondDMSFile`(file) | DesmondDMSFile parses a Desmond DMS (desmond molecular system) and |
| `GromacsGroFile`(file) | GromacsGroFile parses a Gromacs .gro file and constructs a set of atom positions from it. |
| `GromacsTopFile`(file[, periodicBoxVectors, ...]) | GromacsTopFile parses a Gromacs top file and constructs a Topology and (optionally) an OpenMM System from it. |
| `PDBFile`(file[, extraParticleIdentifier]) | PDBFile parses a Protein Data Bank (PDB) file and constructs a Topology and a set of atom positions from it. |
| `PDBxFile`(file) | PDBxFile parses a PDBx/mmCIF file and constructs a Topology and a set of atom positions from it. |

## Representation and Manipulation¶

|  |  |
| --- | --- |
| `Topology`() | Topology stores the topological information about a system. |
| `Modeller`(topology, positions) | Modeller provides tools for editing molecular models, such as adding water or missing hydrogens. |

## Simulation¶

|  |  |
| --- | --- |
| `ForceField`(\*files) | A ForceField constructs OpenMM System objects based on a Topology. |
| `Simulation`(topology, system, integrator[, ...]) | Simulation provides a simplified API for running simulations with OpenMM and reporting results. |

## Reporting Output¶

|  |  |
| --- | --- |
| `CheckpointReporter`(file, reportInterval) | CheckpointReporter saves periodic checkpoints of a simulation. |
| `DCDReporter`(file, reportInterval) | DCDReporter outputs a series of frames from a Simulation to a DCD file. |
| `PDBReporter`(file, reportInterval) | PDBReporter outputs a series of frames from a Simulation to a PDB file. |
| `PDBxReporter`(file, reportInterval) | PDBxReporter outputs a series of frames from a Simulation to a PDBx/mmCIF file. |
| `StateDataReporter`(file, reportInterval[, ...]) | StateDataReporter outputs information about a simulation, such as energy and temperature, to a file. |

## Extras¶

|  |  |
| --- | --- |
| `Atom`(name, element, index, residue, id) | An Atom object represents a residue within a Topology. |
| `Chain`(index, topology, id) | A Chain object represents a chain within a Topology. |
| `CharmmPSFWarning` | For non-fatal PSF parsing issues |
| `CharmmParameterSet`(\*args, \*\*kwargs) | Stores a parameter set defined by CHARMM files. |
| `Element`(number, name, symbol, mass) | An Element represents a chemical element. |
| `Residue`(name, index, chain, id) | A Residue object represents a residue within a Topology. |

High performance molecular simulation on GPUs


### Navigation

- Application Layer
  - Loaders and Setup
  - Representation and Manipulation
  - Simulation
  - Reporting Output
  - Extras
- Library Layer

©2015, Stanford University and the Authors.
|
Powered by Sphinx 1.3.1
& Alabaster 0.7.7
|
Page source
